# Enabling stochastic programming for improved forest planning under uncertainty: moving from theory to practice

**DOI:** 10.1080/02827581.2026.2672350

**Published:** 2026-06-17

**Authors:** Patrik Ulvdal, Mikael Rönnqvist, Göran Ståhl, Ljusk Ola Eriksson, Lars Sängstuvall, Karin Öhman

**Affiliations:** a Department of Forest Resource Management, Swedish University of Agricultural Sciences, Umeå, Sweden; b Forestry Research Institute of Sweden, Ekebo, Sweden; c Département de génie mécanique et de génie industriel, Université Laval, Québec, Canada; d Department of Southern Swedish Forest Research Center, Swedish University of Agricultural Sciences, Alnarp, Sweden; e Bergvik Skog Öst AB, Gävle, Sweden

**Keywords:** Data errors, data quality, decision support system, forest planning, mixed integer programming, stochastic programming, uncertainty

## Abstract

Forest planning faces many uncertainties, yet existing decision support systems (DSS) seldom incorporate techniques to address them. This study explores how stochastic programming (SP) functionality could be added to forest DSSs to account for data uncertainty, aiming to investigate the added value of such functionality. An SP model was applied to a traditional long-term forest planning problem, and its quantitative performance was compared to deterministic optimisation. The user value of the DSS integration was explored in a workshop with potential users. The findings indicate that incorporating SP in a DSS is feasible both user-wise and for quantitatively improving the decisions, even if computational time and model complexity increase. Quantitatively, SP increased the total expected NPV by at least 2% compared to deterministic optimisation. Users involved in evaluating the SP integration acknowledged the benefits of using SP in forest planning, but expressed concerns about the increased complexity in problem specification and results interpretation. To enhance user adoption, the presentation of SP settings and outcomes should be done in a user-friendly manner, including intuitive visualisations and simplified summary statistics. This study underscores the potential of integrating SP in DSSs to improve long-term forest planning under data uncertainty.

## Introduction

Forests provide ecosystem services that can be utilised in many ways (Brockerhoff et al. 2017). The utilisation is generally directed by some decision maker, such as a forest owner, who uses forest planning to generate knowledge on managing a forest according to the decision maker’s aims (Bettinger et al. 2017). The planning process is often aided by optimisation (Kaya et al. 2016). With optimisation, the decision maker describes the planning problem, e.g. what management decisions to make in each stand, as an optimisation model and solves it with an algorithm that generates a solution that maximises or minimises an objective function while fulfilling a set of constraints. Linear programming (LP) has become a standard modelling approach in long-term forest planning (Rönnqvist 2003). However, for many applications in forestry, decisions need to be binary. In those cases, mixed integer programming (MIP) can be used. Whether LP or MIP is used, a key aspect is that the model by design must be deterministic, i.e. all model data must be known with certainty or at least assumed to be. However, complete and certain knowledge is not the common case in forestry. Even so, deterministic optimisation is commonly used in forest planning (Rönnqvist 2003; Ulvdal et al. 2023).

Many types of uncertainty impact forest planning, with forest data uncertainty as one important example (Duvemo and Lämås 2006). Forest data uncertainty affects both our perception of the initial state of forest resources and projections for forest development based on that data, thus impacting decisions about forest management and how implementable they are. Many suggestions for methods that deal with uncertainty have been made (Pasalodos-Tato et al. 2013; Veliz et al. 2014; Labarre et al. 2025), but few have been made easily available as decision-support tools for forestry (de Pellegrin Llorente et al. 2023; Ulvdal et al. 2023).

One promising optimisation method to consider when trying to address data uncertainty is stochastic programming (SP). With SP, the parameters of the optimisation model belong to a probability distribution instead of being a single point value, i.e. the expected value of that distribution (Beale 1955; Dantzig 1955). The distribution is usually represented by a set of scenarios. SP has been applied to forestry contexts in research at least since the 1980s (Gassmann 1989). Early examples of SP studies in forest planning acknowledged the risk of wildfire (Gassmann 1989; Boychuk and Martell 1996). Later, Eriksson (2006) showcased SP with an example that found the maximum net present value (NPV) under climate change-induced growth uncertainty. Future price and growth uncertainty have also been acknowledged (Alonso-Ayuso et al. 2011; Álvarez-Miranda et al. 2019). One study used SP to schedule road maintenance in combination with harvests while accounting for the risk of road work delays (Gomes et al. 2021). Recently, Eyvindson et al. (2024) integrated storm damage risk in tactical planning. In addition, many studies have used SP for creating plans under inventory data or growth model uncertainty (Eyvindson and Kangas 2014; Kangas et al. 2014; Eyvindson et al. 2017; Nahorna et al. 2024). The latter examples show that SP can successfully be used in the context of uncertain forest data.

However, even if SP seems to be a promising method for including consideration of uncertainty, it has not been widely used by practitioners in the forest sector, probably because SP has not been implemented in any forest decision support systems for long-term harvest scheduling (Pasalodos-Tato et al. 2013; de Pellegrin Llorente et al. 2023). A forest decision support system (DSS) incorporates databases of forest data, models describing the development (growth) of the forest according to management, optimisation models defined by the decision maker, solution techniques for the stated problem, and result generation and visualisation (Lämås et al. 2023). This type of DSS is frequently used in forestry (Borges et al. 2014; Segura et al. 2014), e.g. for scheduling harvests. If SP could be implemented in such systems, users would be able to include uncertainty directly in problem-solving and planning. However, an implementation would need to focus on the end-users’ experiences (Schulz 2021). Little would be gained by efforts to improve a DSS to deal with uncertainty if it became too difficult to use or if users did not see any added benefit from the new methodology (Stohr and White 1982).

Therefore, this study’s objective is to explore how SP could be included in a forest DSS to acknowledge forest data uncertainty and to assess the benefits of that functionality for potential users with Swedish forestry as an example. This was achieved by the following steps:

1. developing a long-term forest planning SP model that acknowledges uncertainty in the data describing the initial state of the forest,
2. applying the SP model in a case study showing that the model is implementable in an existing forest DSS and applicable to large, realistic planning problems,
3. providing a quantitative estimate of the benefit (i.e. the increase of objective fulfilment) of acknowledging forest data uncertainty with SP compared to deterministic optimisation, and
4. by involving forest planning experts from the industry as potential users, evaluating the qualitative user benefit and experience of the DSS implementation of the above SP model.

## Materials and methods

### SP model

We developed an SP model expanding the setup of standard deterministic optimisation models used by Swedish forest companies in their strategic planning for finding sustainable harvest levels (Ulvdal et al. 2023). The model is an example of a typical large Nordic forest-owning company with an integrated industry certified according to the Forest Stewardship Council (FSC) scheme. The model aims to find a solution that provides the highest possible NPV while providing a non-declining supply of harvested volume from final fellings. Other restrictions aim to ensure that the solution is implementable according to Swedish law and the FSC scheme. The model is a MIP model due to the need to express some restrictions with binary variables. The main decision variable, i.e. the proportion of each stand to be treated with a certain treatment alternative, is continuous. The model was deliberately designed to be similar to models used in practice. Maintaining structural continuity with standard models allowed comparability with current planning setup, increased transparency for the practitioners involved in the study, and made the case for practical implementability stronger.

An SP model has parameters that belong to continuous distributions, so it is standard procedure to develop its deterministic equivalent to make the model solvable with MIP methodology. Parameters in a deterministic equivalent model are discrete realisations over a set of scenarios that approximate the distributions, which is also the case for the SP model in this study (see section 2.2.1).

The SP model is presented equation-wise below.

(1) $$\begin{array}{rl} & \mathrm{maximise}\;Z_{\mathrm{SP}}\\ & =\frac{1}{\left|S\right|}\left(\underset{s\in S}{\sum }\underset{i\in I}{\sum }\underset{\;j\in J_{i}}{\sum }x_{\mathrm{ij}}n_{\mathrm{sij}}a_{i}-\underset{s\in S}{\sum }\underset{\;p\in P}{\sum }\underset{r\in R}{\sum }\frac{\beta_{\mathrm{rsp}}c_{r}}{1.03^{5p-2.5}}\right) \end{array}$$

Equation (1) is the objective function and states that the objective is to maximise the total expected NPV for all stands in the forest and all scenarios, minus the total discounted penalty for not fulfilling area and harvest levels restrictions. The interest rate for all discounting in the model was set to 3%, to have it as similar as possible to rates used in forest planning in the Nordic countries (Brukas et al. 2001). This approach maintains comparability to the standard approach used by the companies participating in the workshop. This rate was also applied to the penalty terms describing any violation of the ecological requirements, which is motivated by the fact that the penalty cost represents a weighed monetary value. $Z_{\mathrm{SP}}$ is the objective function of the SP model; S is the set of scenarios for different forest developments due to uncertainty in initial forest data; I is the set of stands; $J_{i}$ is the set of treatment alternatives for stand i; P is the set of periods; R is the set of restrictions; $x_{\mathrm{ij}}$ is a variable representing the proportion of stand iassigned to the treatment alternative j; $n_{\mathrm{sij}}$ is the NPV per hectare from forest management in scenario s, in stand i according to treatment alternative *J*; $a_{i}$ is the area of stand *i;* $\beta_{\mathrm{rsp}}$ is a slack variable that takes a positive value (representing an area or volume) for scenario s and period p such that the restriction r is fulfilled; and $c_{r}$ is the cost per unit in $\beta_{\mathrm{rsp}}$ for not fulfilling restriction r. For r=1, i.e. the cost ($c_{r}$) of not reaching the harvest level objectives, the cost was set to 497 SEK m^−3^. This cost corresponds to the average volume-weighted average wood price in Sweden for the years 2019–2024 according to official statistics (Swedish Forest Agency 2025). The cost per hectare for r>1, was set to 3,000 SEK. This cost parameter represents the opportunity cost of corrective measures to avoid potential down-stream cost and certification or compliance risks. The monetary nature of the opportunity costs motivates the application of the same interest rate as for other cash flows. Using the slack variable $\beta_{\mathrm{rsp}}$ across multiple restrictions ensures solvability also if a particular scenario lacks volumes or areas connected to some restrictions and needs to be included to make the model runnable in more cases. Note that the main difference from a similar but deterministic MIP model is the addition of the set S and the index s in many parameters.

The objective function is subjected to the following restrictions:

(2) $$0\leq x_{\mathrm{ij}}\leq 1\;\mathrm{\forall i}\in I,\forall \;j\in J_{i}$$

Equation (2) states that $x_{\mathrm{ij}}$ is a continuous variable between 0 and 1.

(3) $$\beta_{\mathrm{rsp}}\geq 0\;\mathrm{\forall r}\in R,\mathrm{\forall s}\in S,\;\mathrm{\forall p}\in P$$

Equation (3) states that $\beta_{\mathrm{rsp}}$ is a continuous variable larger or equal to 0.

(4) $$y_{\mathrm{sqp}}\in \{0,1\}\;\mathrm{\forall s}\in S,\;\mathrm{\forall p}\in P,\mathrm{\forall q}\in Q$$

Equation (4) states that $y_{\mathrm{sqp}}$ is a binary variable. $y_{\mathrm{sqp}}$ assigns the forest holding to one of the area classes in Q and helps to calculate the allowable annual harvest area decided by Swedish law (see Equations 7, 9, and 10). Q is the set of area classes defined by Swedish law regarding the proportion of the forest that is older than a theoretical rotation age (see Equations 9 and 10).

(5) $$\underset{\;j\in J_{i}}{\sum }x_{\mathrm{ij}}=1\;\mathrm{\forall i}\in I$$

Equation (5) is the maximum area constraint that ascertains the proportions of assigned treatment alternatives in a stand sum to 1 in all stands.

(6) $$\underset{\;j\in J_{i}}{\sum }b_{\mathrm{ij}}x_{\mathrm{ij}}\geq 0.1\;i\in I$$

Equation (6) ensures that at least 10% of the area in each stand is set aside with no management. $b_{\mathrm{ij}}$ is 1 in stand iwith treatment alternative j if the stand is unmanaged in all periods, otherwise 0.

(7) $$\underset{q\in Q}{\sum }y_{\mathrm{sqp}}=1\;\mathrm{\forall s}\in S,\mathrm{\forall p}\in P$$

Equation (7), together with Equation (4), makes sure that only one area class is used in Equations (9 and 10) by forcing the sum of $y_{\mathrm{sqp}}$ to be equal to 1 in each period and scenario.

(8) $$\underset{i\in I}{\sum }\underset{\;j\in J_{i}}{\sum }v_{\mathrm{sijp}}a_{i}x_{\mathrm{ij}}+\beta_{\mathrm{rsp}}\geq \underset{i\in I}{\sum }\underset{\;j\in J_{i}}{\sum }v_{\mathrm{sij}(\;p-1)}a_{i}x_{\mathrm{ij}}\;\mathrm{\forall s}\in S,\mathrm{\forall p}\in P\;\setminus \;\{\;p_{0}\}\;,r=1$$

Equation (8) enforces a non-declining harvest from final fellings in all periods and scenarios. $v_{\mathrm{sijp}}$ is the harvested volume from final fellings per hectare in scenario s, in stand iwith treatment alternative j in period *p*. $p_{0}$ is the first period in P.

(9) $$\underset{i\in I}{\sum }\underset{\;j\in J_{i}}{\sum }d_{\mathrm{ijp}}a_{i}x_{\mathrm{ij}}\leq 5\underset{q\in Q}{\sum }e_{s}f_{q}y_{\mathrm{sqp}}\underset{i\in I}{\sum }a_{i}+\beta_{\mathrm{rsp}}\;\mathrm{\forall s}\in S,\mathrm{\forall p}\in P,\;r=2$$

Equation (9) ensures that the harvested area does not exceed the allowable harvest area according to Swedish law in all periods and scenarios. $d_{\mathrm{ijp}}$ is 1 in stand i with treatment alternative j if the stand is subjected to clear cut in period p, otherwise 0; $e_{s}$ is an area factor from Swedish law, taking the value 0.014 in scenario s if the average site productivity of the forest holding is larger than 8 m^3^ha^−1^year^−1^, 0.011 if it is between 8 and 4 m^3^ha^−1^year^−1^, otherwise 0.009; and $f_{q}$ is a correction factor from Swedish law taking the value 1.4 for q=1, 1.8 for q=2, 2.2 for q=3, 2.8for q=4. Note that the number 5 in Equation (9) turns this annual value into a periodic value since each period is equal to five years.

(10) $$y_{\mathrm{sqp}}g_{q}\leq \frac{\sum_{i\in I}\sum_{\;j\in J_{i}}h_{\mathrm{sijp}}a_{i}x_{\mathrm{ij}}}{\sum_{i\in I}a_{i}}\;\mathrm{\forall s}\in S,\mathrm{\forall p}\in P,\;\forall \;q\in Q$$

Equation (10) calculates the correct $y_{\mathrm{sqp}}$ for a given area proportion of forests older than a theoretical rotation age. $g_{q}$ is an area class proportion from Swedish law taking the value 0 for q=1, 0.26 for q=2, 0.51 for q=3, 0.76 for q=4; $h_{\mathrm{sijp}}$ takes the value 1 in scenario s, in stand iwith treatment alternative j in period p if the mean age of the stand is older than the rotation age, otherwise 0. The rotation age is 70 years if the average site productivity of the forest holding is larger than 8 m^3^ha^−1^year^−1^, 90 years if it is between 8 and 4 m^3^ha^−1^year^−1^, otherwise 110 years.

(11) $$\underset{i\in I}{\sum }\underset{\;j\in J_{i}}{\sum }m_{\mathrm{sijp}}a_{i}x_{\mathrm{ij}}\leq 0.5\underset{i\in I}{\sum }a_{i}+\beta_{\mathrm{rsp}}\;\mathrm{\forall s}\in S,\mathrm{\forall p}\in P\;\setminus \;\{\;p_{0}\},r=3$$

Equation (11) is a restriction that forces the area of forests under the age of 20 years to be less than 50% of the total area in all periods and scenarios (as stipulated by Swedish law). $m_{\mathrm{sijp}}$ is 1 in scenario s, in stand iwith treatment alternative j in period p, if the age of the stand is <20 years, otherwise 0.

(12) $$\underset{i\in I}{\sum }\underset{\;j\in J_{i}}{\sum }\mu_{\mathrm{sijp}}a_{i}x_{\mathrm{ij}}=0\;+\beta_{\mathrm{rsp}}\;\mathrm{\forall s}\in S,\mathrm{\forall p}\in P,\;r=4$$

Equation (12) ensures that illegal harvests in stands that have not reached the legal age limit are not conducted. $\mu_{\mathrm{sijp}}$ takes the value 1 if a final felling is conducted in stand i according to treatment alternative j in scenario s and period p and the age of that stand is lower than the lowest legal final age.

(13) $$\underset{i\in I}{\sum }\underset{\;j\in J_{i}}{\sum }t_{\mathrm{rsijp}}a_{i}x_{\mathrm{ij}}+\beta_{\mathrm{rsp}}\geq u_{r}\underset{i\in I}{\sum }a_{i}\;\mathrm{\forall s}\in S,\mathrm{\forall p}\in P\;\setminus \;\{\;p_{0}\},\;r=\{5,6\}$$

Equation (13) is a combined restriction that for r=5 makes sure that all stands have a proportion of broadleaf trees higher than 10% in all periods and scenarios, and for r=6 makes sure that the area of old forest makes up at least 2% of the total forest area in all periods and scenarios. $t_{\mathrm{rsijp}}$ is 1 in scenario s, in stand iwith treatment alternative j in period p for r=5 if the proportion of broadleaf stems is higher than 0.1, otherwise 0, $t_{\mathrm{rsijp}}$ is 1 in scenario s, in stand iwith treatment alternative j in period p for r=6 if the stand is older than 140, otherwise 0, $u_{r}$ takes the value 1 for r=5 and 0.02 for r=6.

(14) $$\underset{i\in I}{\sum }\underset{\;j\in J_{i}}{\sum }\sigma_{\mathrm{sijp}}a_{i}x_{\mathrm{ij}}+\beta_{\mathrm{rsp}}\geq 0.05\underset{i\in I}{\sum }a_{i}\varphi_{i}\;\mathrm{\forall s}\in S,\mathrm{\forall p}\in P\;\setminus \;\{\;p_{0}\},r=7$$

Equation (14) ensures that the area of broadleaf forest on mesic to moist soils makes up at least 5% of the total mesic to moist forest area in each scenario. $\sigma_{\mathrm{sijp}}$ is 1 in scenario s, in stand iwith treatment alternative j in period p if the stand is dominated by broadleaf trees and the soil is mesic to moist but not wet, otherwise 0 and $\varphi_{i}$ is 1 in stand iif the soil in the stand is mesic to moist but not wet, otherwise 0. Equations (12) to (14) are restrictions connected to the FSC standard (FSC 2020).

### Applying the SP model in a case

**Figure 1.**
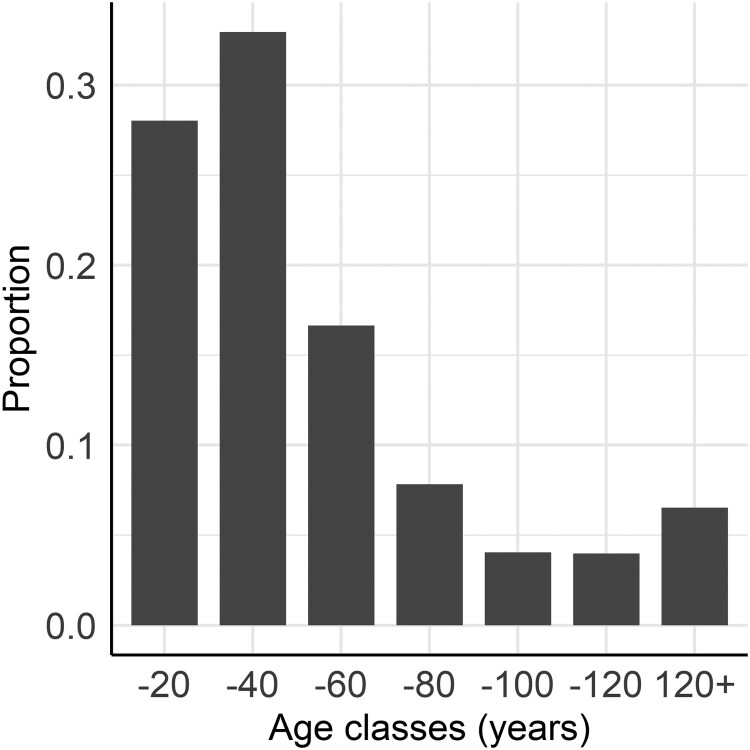
The area class proportions of the forest holding as represented by the input data. The first class (−20) includes bare land.

The SP model (Equations 1–14) was applied to a forest holding in northern Sweden. The forest holding consisted of 23,952 hectares of productive forest land divided into 3,087 stands in northern Sweden (approximately 64°N 19°E). The average growing stock was 102m^3^ha^−1^, and the average stand age was 43 years. The age class distribution was typical for company-owned forests in the area (Figure 1). 56% of the volume consisted of Pinus sylvestris. The rest was 23% Picea abies, 14% Betula spp., and 7% Pinus contorta. The data about the forest holding was organised in a stand inventory with various attributes regarding the tree layer and site qualities for each stand. The inventory data stemmed from various unknown sources and dates. Such inventories in the Nordic countries are generally gathered over time with a mix of subjective field assessments, manual image interpretation, harvester feedback and remote sensing in the form of laser scanning (Ulvdal et al. 2023).

#### Monte Carlo simulation of scenarios

In total, 100 uncertainty scenarios representing discrete realisations of continuous distributions for a set of forest attributes describing the initial state of the forest were generated with the help of Monte Carlo simulation. The attributes that were considered were mean tree diameter at breast height (cm), Lorey’s mean height (m), number of stems (ha^−1^), stand basal area (m^2^ha^−1^), stand mean age (years), and site index (m).

The 100 scenarios of the initial state were simulated independently by generating 10 evenly spaced random vectors from a standard normal distribution for each stand. To make the distributions of the generated deviations multivariate and have similar variances as data in stand inventories, a covariance matrix of deviations between an objective field survey and operational data of lower quality for a set of stands was used in a standard Cholesky decomposition and multiplied with the random vectors (described in, for example, Holmström et al. 2003). Not all stands included in that operation were located in the case area. However, they belonged to the same forest owner in the same part of Sweden.

In the final step, one of the 10 finalised vectors of random but covaried deviations from the mean was randomly picked independently for each stand to form one of the 100 scenarios of the initial state. This step was repeated 100 times. Note that we assumed that the original data for each stand is the mean of a normal distribution, i.e. its expected value. The original stand inventory was kept as scenario 0, resulting in 101 scenarios in total used in the analyses to describe the initial state of the forest. See Appendix A in Ulvdal et al. (2025) for more details regarding the methodology of producing the covariance matrix and generating scenarios.

#### Heureka and generation of treatment programmes

The generation of treatment alternatives, including the forecasting of the development of stands according to 100 scenarios representing the uncertainty regarding the initial state of the forest, was done utilising Heureka PlanWise (2.23.0.2).

Heureka PlanWise is a forest DSS commonly used within the forest sector in Sweden (Lämås et al. 2023). At its core are the models that make forecasts about forest stands given what forest management activities could be performed. The generation of potential activities is guided by user-defined rules and settings grouped as silvicultural strategies and results in treatment programmes (TPs) used as treatment alternatives in optimisation models. A TP is a sequence of silvicultural activities over the planning horizon and the forecasted values from following that sequence, with a time resolution of five years. Thus, Heureka PlanWise follows the Model I formulation, where a TP explicitly covers the whole planning horizon (Johnson and Scheurman 1977). Heureka PlanWise has, at this time, no built-in functionality for considering uncertainty in the form of SP.

The set of alternative TPs, covering a planning horizon of 100 years, comprising 20 periods, was generated for each stand in the original stand inventory, following a silvicultural strategy consistent with Nordic standard rotational forestry. The strategy included one thinning and clear-cutting up to 50 years after the lowest legal age for clear-cutting. Regeneration was done by planting approximately 2,500 conifer seedlings after mechanical soil preparation. At least ten retention trees and three high stumps per hectare were left after thinning and clear-cutting; furthermore, all treatments aimed to leave at least 10% broadleaf trees. Additionally, a no-management alternative was generated for each stand. 13 TPs were generated per stand on average.

The sequence and timing of management activities from these original TPs, i.e. the TPs based on the original data, were extracted and applied to the 100 uncertainty scenarios regarding initial forest data. Thus, the resulting TPs describe the outcome of potential forest management decisions for each stand across all 101 scenarios. These TPs served as treatment alternatives for the optimisation models used in the study.

### The quantitative value of using stochastic programming

The quantitative value of using stochastic programming (VSS) was calculated as the difference between the objective function value gained from solving the SP problem ($Z_{\mathrm{SP}}$) and the expected objective function value ($Z_{\mathrm{EEV}}$) from solving the deterministic version of the same problem, i.e. the expectation of the expected value (EEV) problem (Birge 1982). Formally, this can be stated as.

(15) $$\mathrm{VSS}=Z_{\mathrm{SP}}-Z_{\mathrm{EEV}}$$

To calculate $Z_{\mathrm{EEV}}$, the expected value (EV) problem needed to be solved. The EV problem had parameters that represent the expected values of the uncertain parameters in the SP problem. Thus, the in the EV problem, the setS only included scenario 0, i.e. the original input data that corresponded to the average scenario in the scenario generation. The solution of the EV problem ($x_{\mathrm{ij}}^{\mathrm{EV}}$) was transferred to the SP model (i.e. with all scenarios in the set S) to calculate its expectation, i.e. the $Z_{\mathrm{EEV}}$, across all scenarios.

To address the risk of unfavourable outcomes, two measures of downside risk were calculated for the forest NPV results (refer to Eyvindson and Kangas 2018 for details on risk measures in forest planning). The value at risk (VaR) corresponds to the specific outcome for a certain percentile of the scenarios. The conditional value at risk (CVaR) is instead the average value of the outcomes worse than the VaR and was calculated accordingly.

### Sensitivity analysis

To investigate the stability of the SP model, some sensitivity analyses were run where the model was solved for with varying area penalty costs (0–10,000 SEK ha^−1^) when deviating from restrictions. VSS were calculated for all runs.

### The benefits of stochastic programming for the user

To evaluate the qualitative benefits and user experience of using SP in a DSS, we organised a workshop with representatives from large forest-owning organisations in Sweden. An invitation was sent to all organisations owning more than 200,000 hectares of productive forests in Sweden. Of the six invited organisations, the largest four agreed to participate in the workshop, representing 31% of Sweden's total productive forest area. Each organisation nominated individuals recognised for their forest planning expertise and familiarity with optimisation techniques and the Heureka PlanWise DSS. They had titles like forest planning specialist or analyst, and their work involved presenting suggested actions to decision-makers. These participants were potential users of any new DSS functionality.

The workshop, held in Umeå on the 7th of October 2024, was structured into two distinct parts. In the initial session, we presented a paper-based mock-up illustrating how a problem analogous to that presented in the case study is conventionally solved using standard deterministic optimisation procedures within an unmodified DSS and thus without taking uncertainty into account. The model used was similar to those used in the case study.

This demonstration encompassed the full planning process: data importation, initial data assessment, formulation of management strategies, generation of treatment programmes, construction of the optimisation model, solution of the optimisation problem, and subsequent examination of the results. In parallel with the presentation, a semi-structured discussion was initiated to critically evaluate the limitations of the deterministic optimisation approach, particularly concerning the uncertainty inherent in both data and outcomes.

The second session introduced the SP approach for solving the same planning problem. Again, a paper mock-up was used, but this time to showcase the SP methodology. Notable deviations from the deterministic optimisation process included new procedures for generating uncertain data, modified presentations of the initial state to reflect its uncertainty, adjustments to treatment programme generation across scenarios, the incorporation of additional sets and parameters into the optimisation model, and new techniques for depicting uncertainty in the final results. This session was also accompanied by a discussion focusing on participants’ immediate assessments of the advantages and potential drawbacks of adopting the SP framework over conventional deterministic optimisation.

The discussions during the workshop were guided by a workshop guide (see supplementary material) and digitally recorded to aid later analysis, which focused on finding consensus and non-consensus matters regarding the workshop content. Author PU led the workshop, the discussions, and the following analysis. All other authors except LS participated in the workshop and were actively engaged in the discussion. However, the reported results focus on the external participants’ views, ideas, and statements.

### Optimisation and data processing

The optimisation problems using the TPs generated by Heureka PlanWise were formulated and solved in CPLEX Optimization Studio 22.1.1 with a relative MIP gap tolerance of 1%. The computer had a 3.5 GHz Intel i9-10920X processor with 12 cores and 256 GB of working memory. All other data processing, including creating figures, was done in R 4.4.1 with RStudio 2024.09.1.

## Results

### Case results

**Figure 2.**
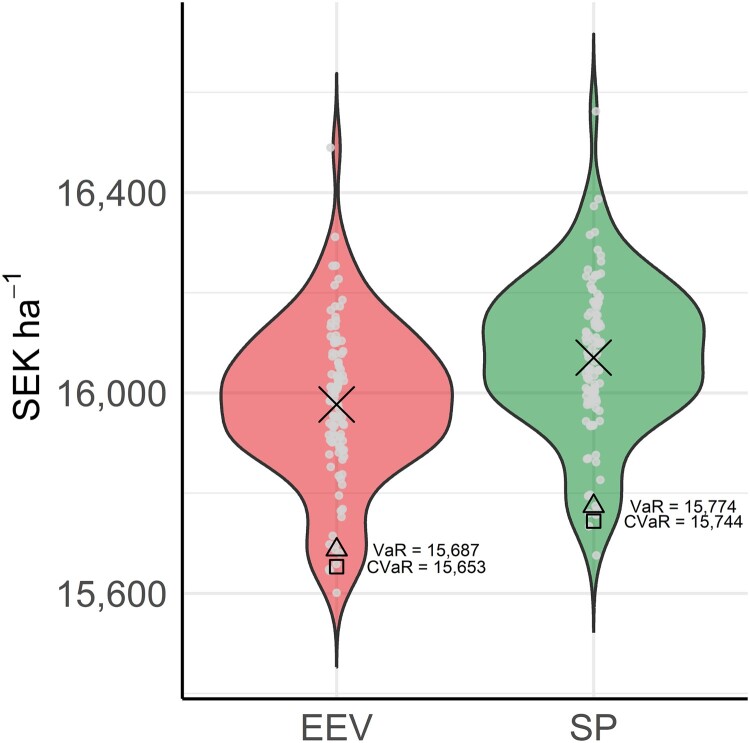
The distribution of the forest management net present value (NPV) in the underlying scenarios for both problems. The means of the distributions are indicated by black crosses. The grey dots are the individual results for the 101 scenarios. The coloured areas are violin plots, showing the density of the grey points as the width of the plot. The triangles indicate the value at risk (VaR), i.e. the NPV of the 5th percentile. The squares indicate the conditional value at risk (CVaR), i.e. the average NPV for all scenarios equal to or less than VaR. EEV is the expectation of the expected value problem and SP is the stochastic programming problem.

The objective of the SP model was to maximise the expected NPV from forest management while minimising the discounted costs of not meeting the restrictions. The expected forest management NPV was higher when acknowledging uncertainty in SP than the opposite with EEV (Figure 2). The VSS, i.e. the increase of the objective function value, was 4.8%, while the forest management NPV increased by 0.6% or 94SEKha^−1^ (Table 1). The large difference between the increase of the objective function value and the forest management NPV is due to the objective function containing penalty components (Equation 1) that make the constraints in the models soft.

**Table 1.** The results for the individual terms of the objective function for the EEV and SP problems.

	EEV	SP	Difference
Z (SEKha^−1^)	8,630	9,045	+4.81%
NPV (SEKha^−1^)	15,977	16,071	+0.59%
Area penalty (SEKha^−1^)	7,044	7,020	−0.34%
Volume penalty (SEKha^−1^)	303	6	−98.16%

The VaR for the 5th percentile increased by 87 SEKha^−1^ when moving from EEV to SP (Figure 2). The corresponding increase for CVaR was 91 SEKha^−1^.

**Figure 3.**
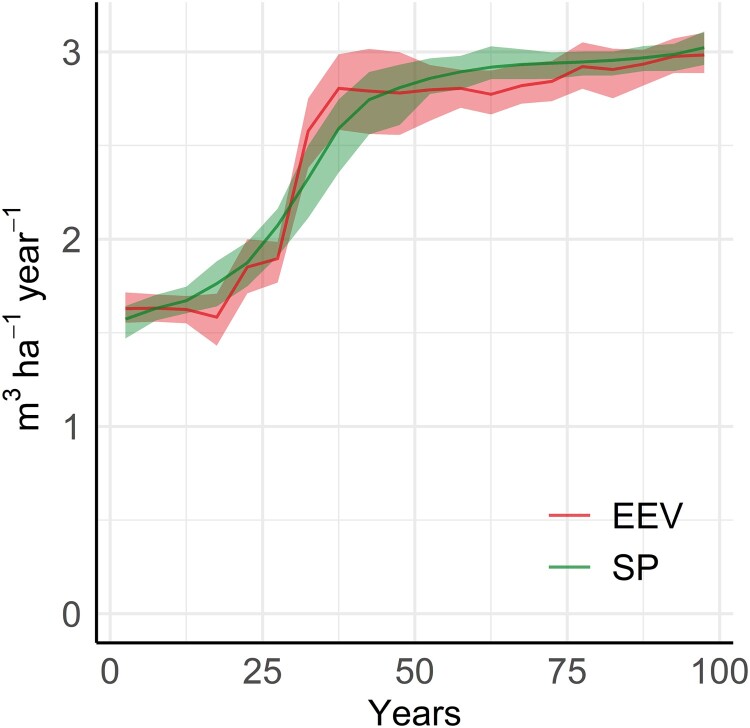
The expected values for total harvest levels from final fellings are shown as coloured lines. The full extents of all underlying scenarios are shown with coloured areas. EEV is the expectation of the expected value problem and SP is the stochastic programming problem.

The model was restricted from decreasing harvest levels from final fellings over time. On the average, this was achieved in both SP, while EEV saw some periods of declining levels (Figure 3). However, since the total volume penalty was larger than 0 for SP, some scenarios must have had a small decrease even in SP (Table 1). Harvest levels from final fellings increased steadily during the first 40–50 years in SP and less steadily in EEV (Figure 3). The SP model found a solution that provided higher harvest levels during multiple periods at the beginning of the planning horizon, thus contributing to the higher NPV. The width of possible outcomes, i.e. uncertainty, was generally larger for EEV than SP during multiple periods.

**Figure 4.**
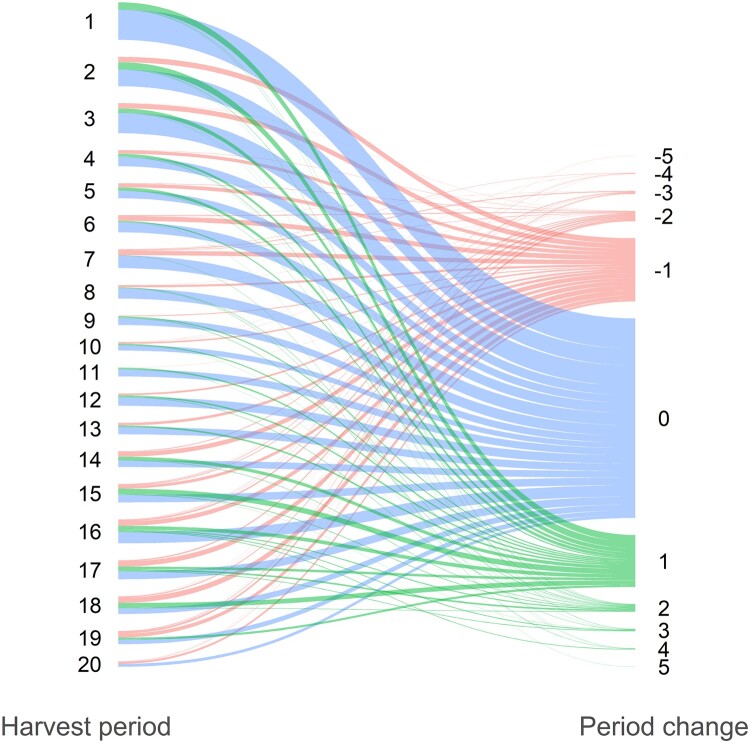
The relative change in timing in periods (5 years) for first final felling for each stand in EEV compared to SP. Blue colour indicates that no change occurred in timing of the first final felling, red colour that it was advanced, and green that it was postponed.

Regarding changes in actual management, we looked at changes during the first period. If the timing was changed, more harvests were postponed than advanced (Figure 4). The most common change was to postpone or advance by only one period. Furthermore, it was more common for stands that had their first final felling early during the planning horizon to remain stable than for those with final fellings later.

**Figure 5.**
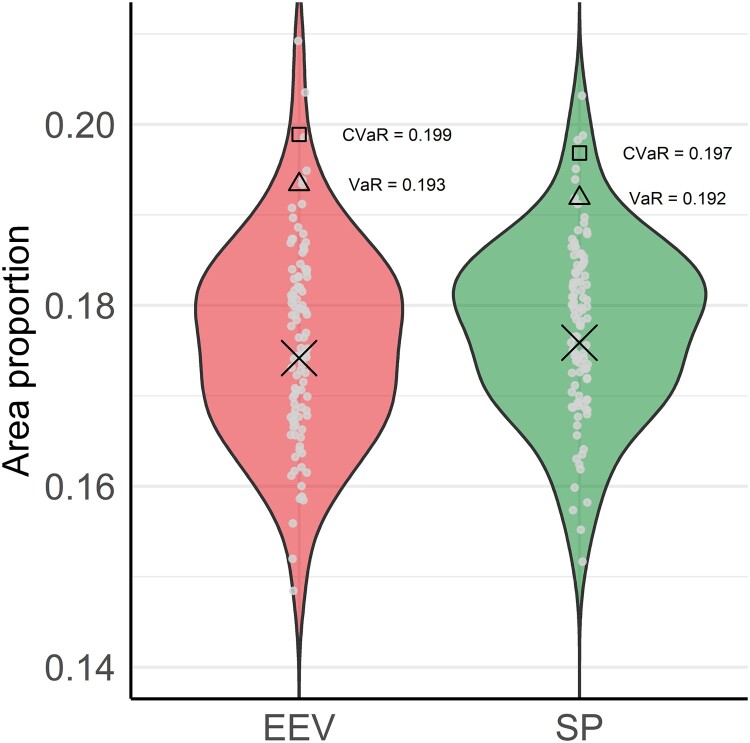
The distribution of the average area share of illegal harvests per period in the underlying scenarios for both problems. The means of the distributions are indicated by black crosses. The grey dots are the individual results for the 101 scenarios. The coloured areas are violin plots, showing the density of the grey points as the width of the plot. The triangles indicate the value at risk (VaR), i.e. the area proportion of the 95th percentile. The squares indicate the conditional value at risk (CVaR), i.e. the average area proportion for all scenarios equal to or larger than VaR. EEV is the expectation of the expected value problem and SP is the stochastic programming problem.

To increase solvability, our model allowed that harvests could be performed earlier than the legal requirement. This resulted in an average area share of illegal harvests per period of around 18% (Figure 5). Both the VaR and CVaR for the level of illegality were improved with SP (Figure 5). The average area-weighted age difference between the actual harvest and the legal requirement was −9.2 years for the illegal harvests, and +16.4 years for the legal harvests, resulting in a total average age difference of +11.9 years.

Additional results regarding other indicators, such as harvested assortments, harvest diameters, harvest age, area developments, and indicators for biodiversity from the case, are presented in Appendix A.

The SP problem had 48,115 constraints and 80,777 variables. It took 1,452 s to solve, resulting in a MIP gap of 0.002%. The EV problem, whose solution was used to calculate EEV, had 6,615 constraints and 40,877 variables and took 8 s to solve with a MIP gap of 0.002%. The solution time increased by 1,444 s, or 18,963%, for SP compared to EV.

### Sensitivity analysis results

**Figure 6.**
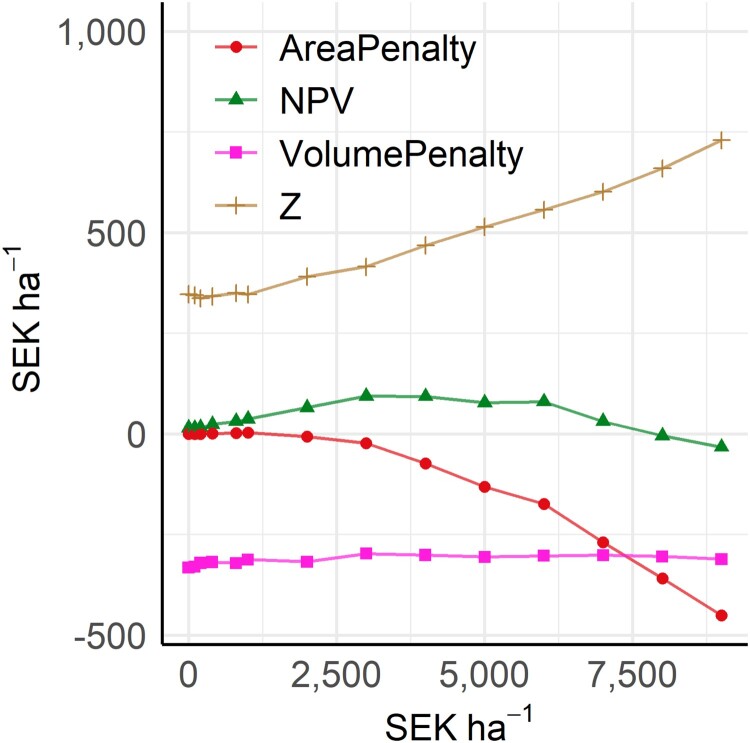
The change in SEKha^−1^for each individual term (y-axis) in the objective function between EEV and SP with increasing area penalty (x.axis) across the runs of the sensitivity analysis. NPV refers to the net present value from forest management, Z to the total objective function value.

**Figure 7.**
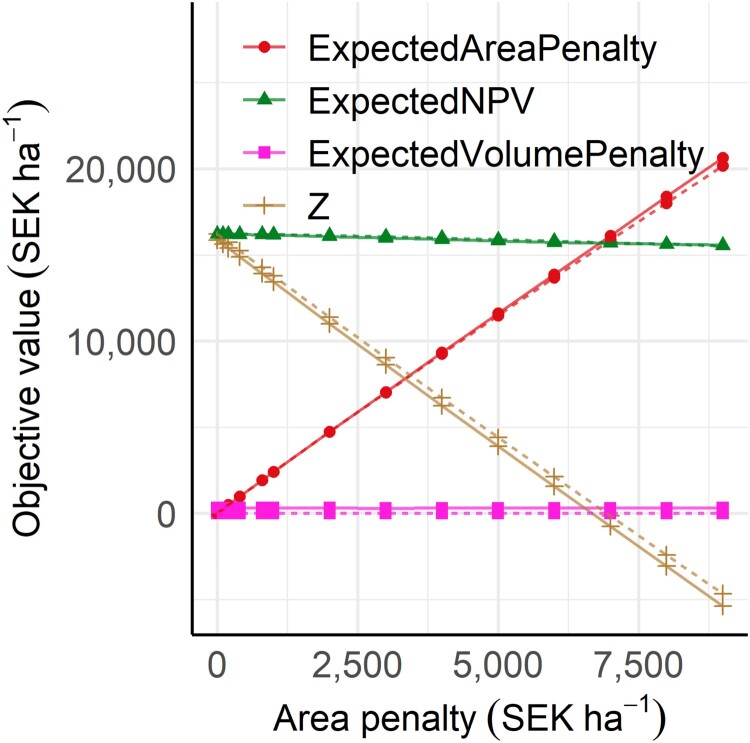
The values in SEKha^−1^ for each individual term (y-axis) in the objective function with increasing area penalty (x-axis) across the runs of the sensitivity analysis. The solid lines show the EEV-solution, the dashed lines the SP-solution. NPV refers to the net present value from forest management, Z to the total objective function value.

The sensitivity analyses showed that VSS (change in Z) increased with increasing area penalty (Figure 6). Also, the change in forest management NPV increased up to the penalty of 3,000 SEK ha^−1^ (as used in the case), but then decreased. The total savings of costs due to the area penalty increased (i.e. the negative values became larger) with increasing penalty levels, while the volume penalty remained relatively stable. The patterns of the VSS are explained by the change in absolute values (Figure 7).

### The user perspective on stochastic programming

The results from the workshop on user experiences and the benefits of using SP in a DSS are divided into four themes. First, we present what the participants discussed regarding uncertainty in their planning processes today and whether it is important to consider or not. Second, we report what they thought about the DSS’s usability today and how it would be affected after a potential implementation of SP. Third, we present the participants’ discussions regarding the presentation of results, including or excluding uncertainties. Last, we present the participants’ views on the benefits of implementing SP in the DSS. A summary of the workshop participants’ stated strengths and weaknesses for standard deterministic MIP versus SP from their perspective as users of a decision support system is presented in Table 2. The DSS used as an example was Heureka PlanWise.

**Table 2.** A summary of the workshop participants’ stated strengths and weaknesses for standard deterministic optimisation versus stochastic programming (SP) from their perspective as users of a decision support system.

	Deterministic	Stochastic
Potential to address uncertainty in data, from models and risk.	–	+
Potential to visualise uncertainty in initial data.	–	+
Potential to visualise uncertainty in results.	–	+
Level of complexity of the model.	+	–
Potential to isolate what factors affect the result.	+	–
Potential to acknowledge catastrophic events.	–	+
Potential to achieve targets with a user-set probability.	-	+
Need for pedagogical explanations and examples.	+	-
Need of knowledge to use.	+	-

Note: A (+) indicates that the method in question provides benefits for the user in that specific regard, a (-) indicates the opposite.

#### Uncertainty in the forest planning process today

Uncertainty is an important topic for forest companies. Overall, there was consensus that uncertainties in forest inventories and model-based forecasts significantly influence planning outcomes. However, other factors, such as the unpredictable effects of climate change on forest growth and the variable harvestability of stands when unexpected natural values emerge, can be even more influential.

One participant described the need to be able to present the uncertainty of future harvest levels to decision-makers. A way of doing that used at that company today was a routine where the quality of a field survey, used as the basis for strategic planning, was extrapolated to estimate the overall reliability of a long-term plan. By doing so, the future harvest levels could be presented with error bars. Although not formally rigorous, this method helped communicate uncertainties, such as those related to proposed harvest levels, to decision-makers.

Two key datasets also illustrated uncertainties that affect planning: a forest resource map based on laser scanning and the operationally used inventories.

All organisations relied on predictions from a publicly available forest resource map developed with airborne laser scanning (Nilsson et al. 2017). While generally well regarded, one participant reported that using these predictions resulted in lower estimates of total standing volume, sparking internal debate.

Several participants highlighted errors in stand age data from operational inventories. Although the age of a planted forest is usually assumed to equal the time since planting, legal criteria for final felling depend on the basal-area weighted mean age, a value that, for example, can be reduced by rapid growth of naturally established seedlings. This mismatch complicates the alignment of long- and short-term plans, as stands deemed mature in strategic plans may, upon field inspection, fall below the required age threshold, disrupting the wood supply chain.

#### The usability of the DSS

During the workshop, participants reviewed the DSS’s usability in its current state and after it being modified to incorporate SP.

Currently, after importing forest data, users can examine an overview of the initial forest conditions with statistics like age and productivity distributions, which mainly serve as a data validation tool. Participants expressed a desire for additional information on the uncertainty of these initial conditions, though they acknowledged that estimating such uncertainty is challenging without dedicated field surveys.

After being introduced to SP, participants suggested using simplified measures (e.g. mean, maximum, and minimum values) and boxplots to present the initial states across scenarios. They also emphasised the need for user-friendly interfaces to examine both individual and aggregated scenarios.

Additionally, participants stressed that a modified DSS must include robust, well-justified default settings, particularly for scenario generation, as many users will rely on defaults, given the complexity of the process. They also noted that it must be clear how illegal treatments and treatments that cannot be conducted in all scenarios are handled; for instance, a final felling might be legal in one scenario but not in another due to uncertainties in the age data.

It was agreed that including scenarios as a set in the optimisation model made it more complex.

#### Presentation of outputs

The participants emphasised the importance of accounting for uncertainty when presenting DSS analyses to decision-makers, often noting, *We do not know exactly, but the result is probably somewhere around here.* Thus, the participants preferred that results be shown as probability distributions, such as a line for the average with accompanying minimum and maximum bounds, instead of single-point estimates. However, some speculated that financial markets might favour simpler figures that did not show any uncertainty.

Participants also found it challenging to intuitively understand how EEV related to SP, predicting that non-expert decision-makers would have even more difficulty. Nonetheless, as expert analysts, they acknowledged that they possess expertise that justifies the complexity and would require less assistance. Overall, they agreed that any interpretation of SP results must consider the methods used to generate the scenarios, making this a crucial aspect of SP implementation in the DSS.

#### The benefits of SP

Participants agreed that although incorporating SP increases the DSS’s complexity, it added clear value to the planning process. One participant noted that there were no apparent *downsides for me as an analyst* and anticipated that SP would enhance decision-making, even if the focus of the initial implementation would solely be on the uncertainty of input data. Some highlighted SP’s potential for tasks like valuing biological assets, while others pointed out that even a small uncertainty spread is informative for risk-sensitive decisions.

Despite one suggestion to add uncertainty-handling methods other than SP, consensus emerged that it is not evident that better alternatives exist. Robust programming was considered but dismissed due to the assumed high costs of solutions in forestry implementations. Another proposed approach was adjusting coefficients directly in the optimisation model by setting minimum and maximum parameter values instead of generating multiple uncertainty scenarios. This method was deemed to be less thorough compared to SP.

Participants also noted that even a basic SP implementation could stimulate new research questions in forest planning and drive the development of improved uncertainty tools. They also stressed the importance of clear, pedagogical explanations for the new methodology, given that the DSS already demands significant user expertise.

## Discussion

In this study, we have shown that the implementation of SP technology in a DSS both quantitatively improves decisions and provides other values for users when explicitly accounting for data uncertainty, a factor often neglected in traditional deterministic models and DSSs.

Quantitatively, our analysis yielded a VSS of approximately 5%, with a corresponding forest management NPV increase of 0.6% (Table 1). These figures are comparable to those reported in other forestry applications of SP, where VSS values have ranged from 0.1% to 20% depending on risk preferences and penalty cost structures (Boychuk and Martell 1996; Hultqvist and Olsson 2004; Alonso-Ayuso et al. 2011; Eyvindson and Cheng 2016). However, one should note that VSS heavily depends on the problem formulation, especially the size of penalty costs in the recourse component of the objective function (Figure 6). Unsurprisingly, the sensitivity analyses showed that VSS changed with increasing area penalty costs. The analysis further showed that the higher a decision-maker sets the area penalty, the higher the increase in total VSS he or she will achieve. The VSS represents a trade-off from forest management NPV, since the positive change of NPV decreases after 3,000 SEK ha^−1^ in area penalty, and becomes negative after 8,000 SEK ha^−1^.

Our qualitative evaluation of SP as a potential new solution technique from the user perspective showed promising results. The integration of SP into forest DSS was seen as a valuable advancement because it would allow for a more nuanced and realistic handling of uncertainty compared to today. The SP framework can lead to more informed and risk-aware forest management decisions by explicitly modelling data and forecasting uncertainties. The workshop participants were generally positive about SP, especially after a thorough explanation. They found the new functionality of showing forecasted developments as distributions rather than point estimates very helpful, especially when explaining the uncertainty of a solution to a decision-maker. It seems that having only that new functionality would add significant value. The workshop results made it clear that the approach with keeping the new SP model similar in structure to the standard formulations used in practice was beneficial for potential user adoption and user experience. However, it should be noted that this somewhat constrained modelling approach does not fully utilise the expressive power of more advanced SP formulations.

From an implementation perspective, integrating SP into systems such as Heureka PlanWise is technologically feasible. The additional functionality that needs to be developed revolves around how to generate uncertainty scenarios of the initial forest state based on the data a user provides, to consider those scenarios in the generation of treatment programmes, to utilise the scenarios as a new set in the optimisation module, and to present the results over the scenarios clearly and understandably. The main challenge would probably be to decide how the scenario generation should work, since this can be done in multiple ways. The scenario generation process must be general and correct enough to be relevant for many users while customisable enough to meet specific needs for specific questions. Preferably, the generation should also be understandable for users. In fact, the latter is important for all development needed for implementing SP, since a new technique like SP introduces more complexity and abstraction to the decision process. What help will a user have from SP if the results are not presented understandably (Schulz 2021)? As pointed out during the early days of DSS development and user experience research, *we need to make the interface functionally useful and enhance productivity and enjoyment of users*; otherwise, no one will use the system (Stohr and White 1982). The fear of introducing complexity can be one reason why there are no general forest DSSs where SP has been implemented available for a broader audience of forest planners (Pasalodos-Tato et al. 2013; de Pellegrin Llorente et al. 2023). The most important aspects to consider before a potential implementation of SP in Heureka PlanWise would be technical aspects regarding the generation of uncertainty scenarios and overall user-friendliness. When defining a module for scenario generation, much effort must be put into finding relevant and justified procedures. Many users will probably not change default settings, as doing so would require considerable technical expertise. The user-friendliness revolves much around providing good examples, easily understandable instructions, and an intuitive presentation of results. Also, the user interface is important in future user adoption, especially if the DSS is aimed towards non-experts (Power and Sharda 2007). Adding SP functionality to the DSS should lead the way for the consideration of other influential uncertainties, like the effects of the ongoing climate change on future growth conditions and damage risks (Usbeck et al. 2010; Lindner et al. 2014; Goude et al. 2022). One important drawback of SP when implementing it in DSS, is increased solution times. In our case, the solution time increased by 18,963%, which would pose problems in real-world applications.

Our SP model resolved the planning problem by employing a recourse component (i.e. summing deviation penalties via slack variables) to soften restrictions across scenarios and to provide information for users of possible infeasibilities. This approach was deliberately chosen to maintain clarity for end users and decision-makers, in line with previous observations that increased model complexity can deter the use of decision-support tools (McIntosh et al. 2011). Furthermore, this penalty approach was needed since SP, in general, needs to be employed with soft constraints; otherwise, some scenarios could have rendered the problem infeasible. As familiarity with SP grows, there is considerable scope for adopting more sophisticated formulations that capture risk, as proposed by, for example, Eyvindson and Cheng (2016). They included a CVaR-component directly in the objective function to acknowledge risk, which is a sound approach, but we deemed it too complex for this first step of moving SP into practice. Anyhow, the results show that the downside risks in our analyses measured with both VaR and CVaR improve with SP compared with the more ignorant case of EEV (Figure 2). Thus, even without explicit risk components, the SP model improves the downside risk. This is an expected result, since SP improves the average outcome and thus improves the expected value of the results.

The incorporation of SP altered management decisions in individual stands. Our results (Figure 4) indicate that final fellings to some extent were rescheduled, either advanced or postponed. This behaviour appears to be a deliberate risk mitigation strategy to avoid costs from erroneous management actions.

The share of illegal harvests (approximately 18%) was rather high (Figure 5). This result might pose a challenge for plan implementation and highlights a potential risk of not being able to follow the law in all scenarios. However, the average shortening of the rotation period was 9 years in the illegal cases, and in total, the rotation periods were on average prolonged by 12 years; thus, more stands had their rotation period prolonged than shortened. In a real implementation phase, the age of the stands would have to be checked thoroughly to avoid illegal activities. Notably, the downside risk (VaR and CVaR) for the share of potentially illegal harvests was decreased with SP (Figure 5).

Although our model employed only 101 scenarios, prior work (Eyvindson and Kangas 2016) suggests that even as few as 10–15 scenarios can reduce the optimality gap to below 1% under risk-neutral conditions, implying that our scenario set is sufficient for capturing the underlying uncertainty. In addition, increasing the number of scenarios will increase the model size, requiring more model generation and solution time. To improve the solvability for larger cases, the SP can be solved using methods like Benders and Lagrangian decomposition (Li and Grossmann 2021). Such methods produce near-optimal solutions (without guarantee) in shorter and more reasonable times for large cases.

Regarding uncertainty generation, it was assumed that a given value for an attribute in a stand in the operational stand inventory was the expected value of a real distribution for that value in that stand. This is a simplification since the value in the stand inventory, in fact, is a realisation of some unknown probability distribution around the true state and thus contains both random and systematic errors. The data generated in the case study thus represents something other than the true distribution of the initial state. We hypothesise that a Bayesian approach, where the values in the stand inventory are used to estimate a true distribution, would be an interesting solution to this problem. That investigation, however, was outside the scope of this study. The results presented in this study are valid, assuming that the original data represent the true mean.

The 3% discount rate follows Nordic forest industry practice (Brukas et al. 2001), however, this approach warrants reflection. First, while SP can incorporate uncertain discount rates (Peymankar et al. 2021), doing so would require modelling stakeholder preferences. Second, ecological penalty costs are discounted at the same rate as monetary cash flows, which could imply reduced concern for future ecological conditions. In practice, however, long-term forest plans are revised every 5–10 years (Nilsson et al. 2012; Ulvdal et al. 2023), meaning ecological constraints would be reassessed regularly. Thus, the discount rate mainly weights decisions within each actionable planning period rather than signalling declining ecological value. Third, discounting applies only to monetary quantities (NPV and penalties). This style of modelling, where deviations from ecological requirements incur discounted opportunity costs, which in turn might be seen as costs for compensatory actions, was suitable for this study’s set up. Recently, other approaches that discount no-monetary values have been presented (Jarisch et al. 2022). For this style of modelling however, it is not evident what best-practice is.

## Conclusion

Overall, the findings indicate that while it would be possible to incorporate SP into a forest DSS, it would introduce added levels of complexity and increased computational burden. However, the quantitative value of considering uncertainty in forest planning should not be neglected. Moreover, the qualitative benefits, particularly in improving uncertainty communication and enabling more robust planning, make SP a promising potential method. For users, the complexity could be reduced by providing intuitive visualisations, simplified summary statistics, and comprehensive documentation with pedagogical examples.

We believe that a modern forest DSS should help users understand the uncertainty of any forecasts and results made with it, but should preferably also help users consider uncertainty in the actual planning process. SP would make it possible to solve both needs. However, when implementing SP, the development should not stop at dealing with uncertain data. Support for dealing with other uncertainties, such as climate change-induced growth responses and increased risk for storm damages, should also be included.

## Figures and Tables

**Figure A1.**
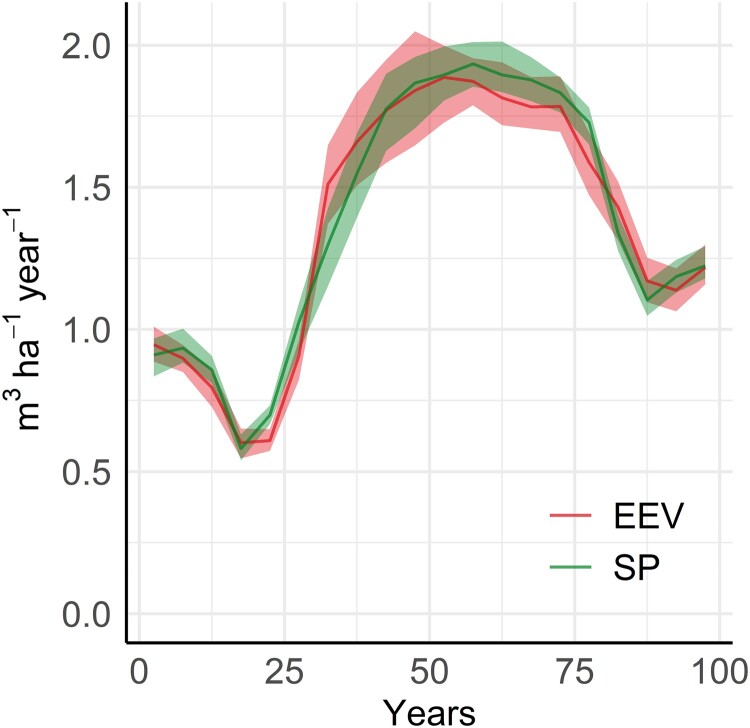
The expected values for harvested timbers are shown as coloured lines. The full extents of all underlying scenarios are shown with coloured areas. EEV is the expectation of the expected value problem and SP is the stochastic programming problem.

**Figure A2.**
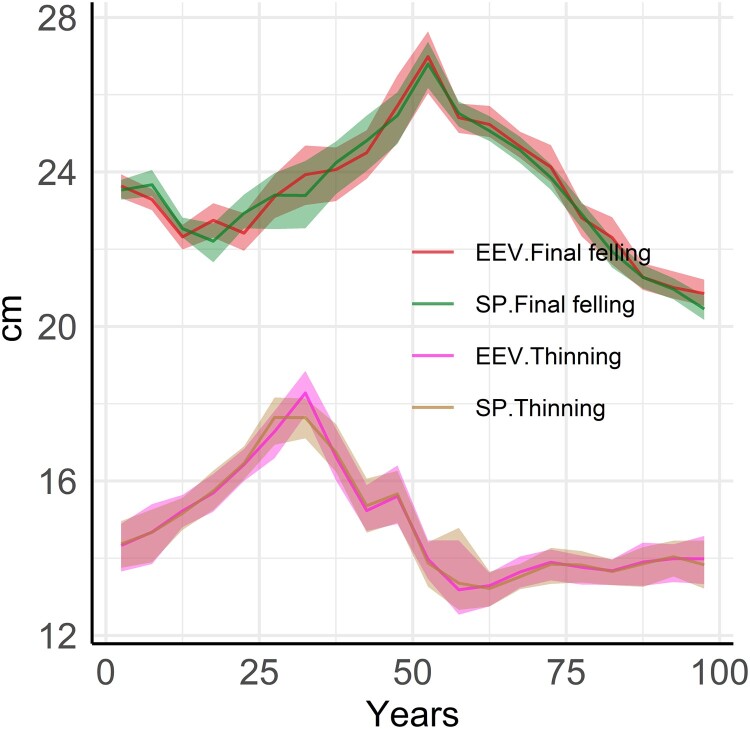
The expected values for average tree diameters in harvests are shown as coloured lines. The full extents of all underlying scenarios are shown with coloured areas. EEV is the expectation of the expected value problem and SP is the stochastic programming problem.

**Figure A3.**
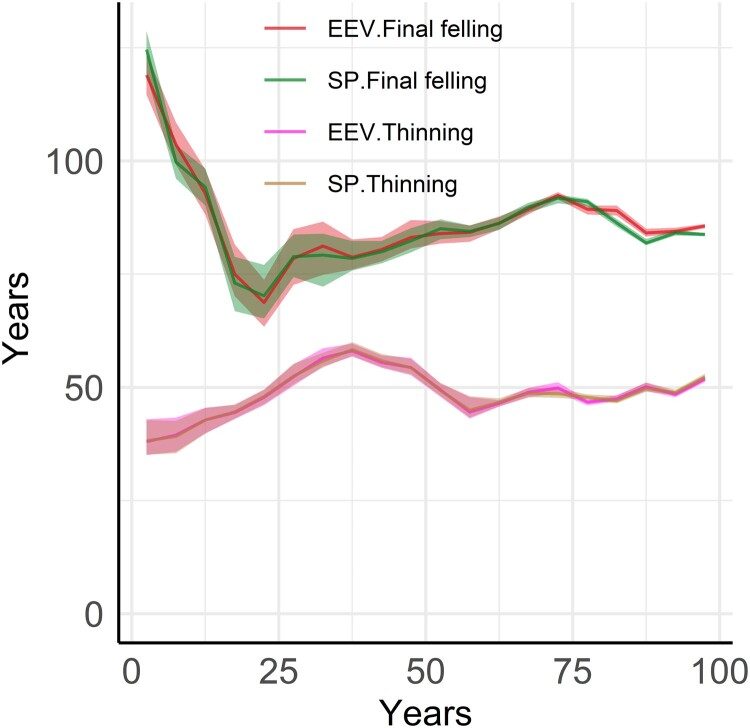
The expected values for age in harvests are shown as coloured lines. The full extents of all underlying scenarios are shown with coloured areas. EEV is the expectation of the expected value problem and SP is the stochastic programming problem.

**Figure A4.**
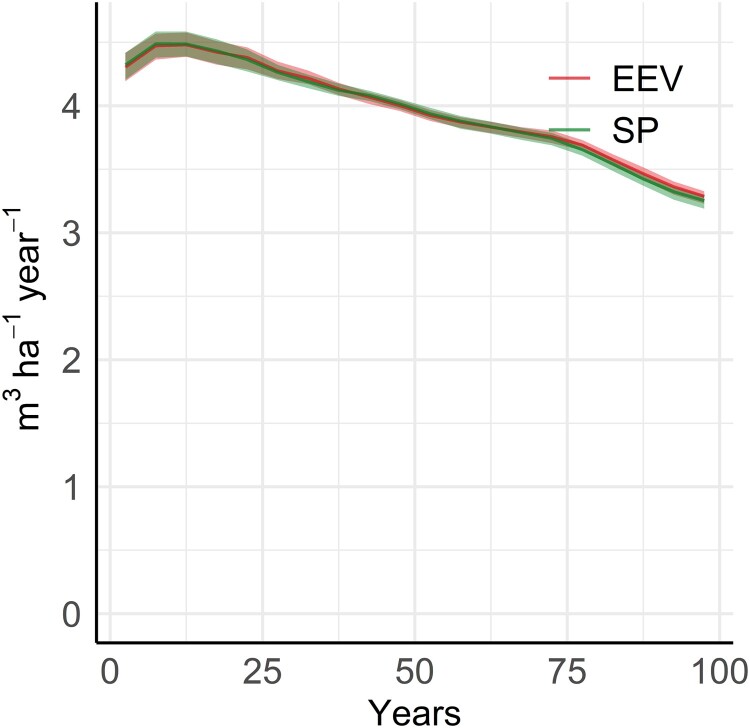
The expected values for net growth are shown as coloured lines. The full extents of all underlying scenarios are shown with coloured areas. EEV is the expectation of the expected value problem and SP is the stochastic programming problem.

**Figure A5.**
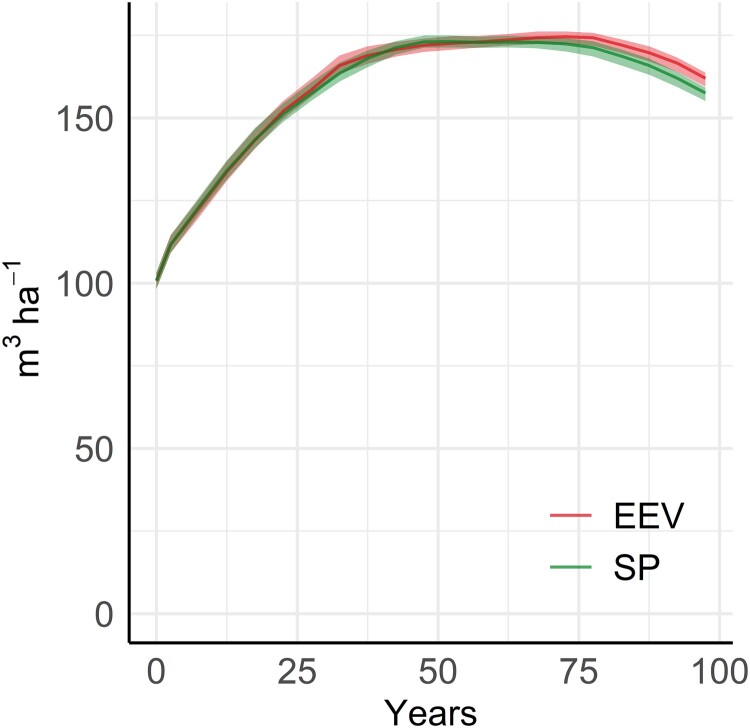
The expected values for total standing are shown as coloured lines. The full extents of all underlying scenarios are shown with coloured areas. EEV is the expectation of the expected value problem and SP is the stochastic programming problem.

**Figure B6.**
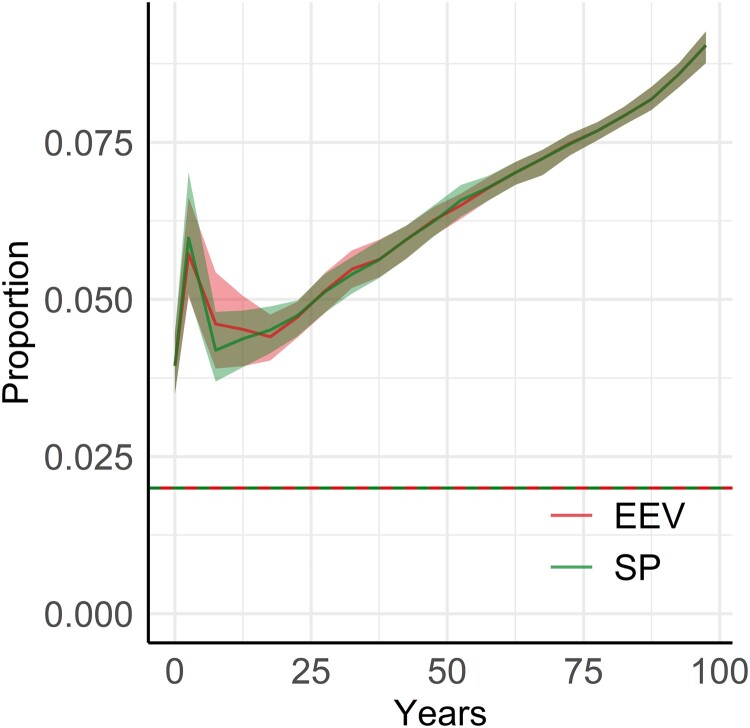
The expected values for the area proportion of forest >140 years old are shown as coloured lines. The full extents of all underlying scenarios are shown with coloured areas. The dashed lines indicated the lowest allowed area proportion (2%). EEV is the expectation of the expected value problem and SP is the stochastic programming problem.

**Figure A7.**
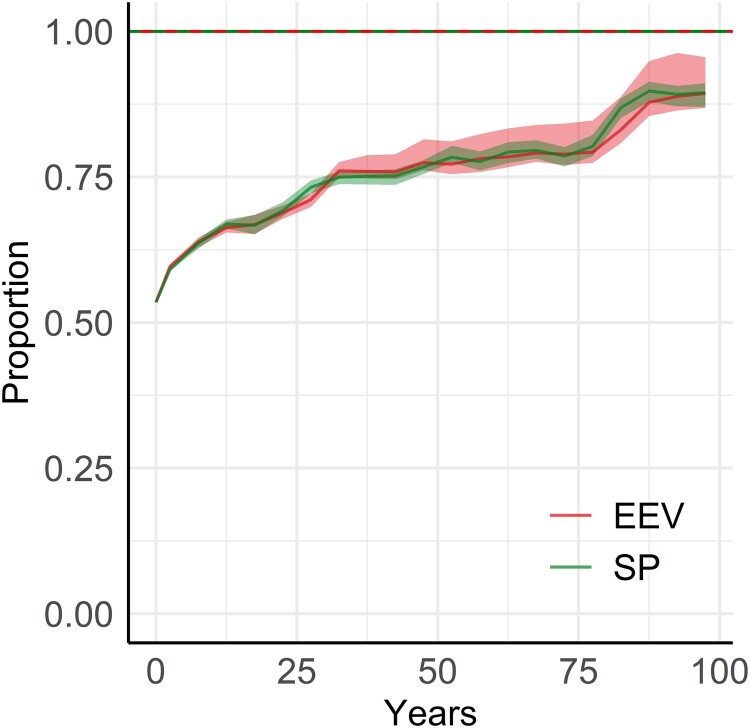
The expected values for the area proportion of forest >10% broad-leaf trees are shown as coloured lines. The full extents of all underlying scenarios are shown with coloured areas. The dashed lines indicated the lowest allowed area proportion (100%). EEV is the expectation of the expected value problem and SP is the stochastic programming problem.

## Data Availability

The original stand inventory and field survey data analysed during this study are unavailable since the data belong to a third party (Holmen Skog AB) and may have financial implications. The workshop guide is available as supplementary material online.

## Code availability

Data processing and optimisation scripts in R, CPLEX and the Heureka Project-file are available from the corresponding author upon reasonable request.

## Appendices

### Appendix A. Additional figures

Harvested timber (Figure A1) deviated more between EEV and SP than the final felling harvest levels (Figure 3), especially in the plan's early years. This indicates that SP could make better choices for maximising harvest value, i.e. harvesting more timber than EEV.

The average diameter of harvested trees impacts profitability and, thus, the NPV. The development of average diameter showed little difference between EEV and SP, especially for thinning (Figure A2). There were larger differences for diameters in final fellings, especially in the far future.

The development of the average age of harvested stands showed that SP harvests somewhat older stands further in time than EEV, indicating that EV, to some extent, selected too young stands for harvests, at least in the earlier years (Figure A3). The development of average age in thinnings was almost identical between EEV and EV.

Net growth showed similar patterns between SP and EEV up until year 50 (Figure A5). The declining trend is a consequence of the age class distribution with a lot of young forests that grew fast at the beginning of the plan but matured later (Figure 1).

Also, the total standing volume showed similar patterns between SP and EEV up until year 50 (Figure A5). The development of the standing volume is a consequence of harvest levels (Figure 3) and net growth (Figure A4) and is also explained by the initial age class distribution (Figure 1).

The model had restrictions regarding the allowed area proportion of old forests (2% > 140 years). This proved not to be a problem to reach (Figure A6). The development of old forests was very similar between SP and EEV, probably because the restriction was relatively easy to fulfil.

Furthermore, the model had restrictions that 100% of all stands should have >10% broadleaf stems. This proved challenging to reach. However, the penalty variable in the objective function, in essence, turned the model into a goal programming problem that strived to reach the target of 100%. Figure A7 shows that an increase in the area of forests with >10% broadleaf trees was possible, but that EEV has much larger uncertainty at the end of the planning horizon.
